# The process of ratifying the treaty to establish the African Medicines Agency: perspectives of national regulatory agencies

**DOI:** 10.1093/heapol/czae017

**Published:** 2024-03-18

**Authors:** Bakani Mark Ncube, Admire Dube, Kim Ward

**Affiliations:** School of Pharmacy, University of the Western Cape, Private Bag X17, Bellville 7535, South Africa; School of Pharmacy, University of the Western Cape, Private Bag X17, Bellville 7535, South Africa; School of Pharmacy, University of the Western Cape, Private Bag X17, Bellville 7535, South Africa

**Keywords:** Medicines regulatory harmonization, African Medicines Regulatory Harmonization initiative, treaty ratification, treaty for the establishment of the African Medicines Agency, African Medicines Agency

## Abstract

The vision of the African Medicines Agency (AMA) is to ensure that all Africans have access to affordable medical products that meet internationally recognized standards of quality, safety and efficacy for priority diseases/conditions. The AMA is being established by a treaty which had to be ratified by a minimum of 15 African countries. Although there was no deadline, the ratification process has been slower than expected. This study therefore analysed the rationale, perceived benefits, enabling factors and challenges of the AMA’s establishment. This study was a qualitative, cross-sectional, census survey of the national medicines regulatory authorities (NRAs) of 45 African countries. The Heads of NRAs and a senior NRA staff member were contacted to complete self-administered questionnaires. The existence of mature NRAs, the desire to have harmonized regulatory systems, the presence of strong political will and appropriate advocacy to expedite treaty signing are all enabling factors for AMA treaty signing. The challenges reported include the fact that the process is slow and there is limited understanding of the process. Competing national priorities, changes in office bearers in the public system and stagnation of the process at the ministerial level were also challenges reported. This study has improved the understanding of the treaty signing and ratification process and the perceived benefits and enabling factors of signing and ratification from African NRAs’ perspective. NRAs also highlighted challenges encountered in the process. Addressing these challenges will result in effective medicines regulation by galvanizing technical support, regulatory expertise and resources at a continental level.

Key messages

- The vision of the African Medicines Agency (AMA) is to ensure that all Africans have access to affordable medical products that meet internationally recognized standards of quality, safety and efficacy for priority diseases/conditions.
- The AMA is being established by a treaty which had to be ratified by a minimum of 15 African countries. Although there was no deadline, the ratification process has been slower than expected.
- The existence of mature national medicines regulatory authorities, the desire to have harmonized regulatory systems, the presence of strong political will and appropriate advocacy to expedite treaty signing are all enabling factors for AMA treaty signing.
- The challenges faced in treaty signing and ratification include the fact that the process is slow, there is limited understanding of the process, there are competing national priorities and changes in office bearers in the public system as well as stagnation of the process at the ministerial level.

## Introduction

Intended to be an African Union (AU) organ (African Union; African Union, 2017), the African Medicines Agency (AMA) has a vision to ensure that all Africans have access to affordable medical products that meet internationally recognized standards of quality, safety and efficacy, for priority diseases/conditions (African Union; 2019a; African Union, World Health Organization, 2014; African Union Commission, World Health Organization, 2014; Schlesinger, 2019). Medical products mean medicines, vaccines, blood and blood products, diagnostics and medical devices (African Union, 2019b). The objectives of the AMA include to coordinate national and subregional medicines regulatory systems, provide robust regulatory oversight and monitor the safety of medical products to decrease the circulation of substandard and falsified (SF) medical products, and to promote international cooperation, partnerships and harmonization of technical requirements (African Union). The vision and objectives of the AMA will be achieved through pooling expertise and capacities available in Africa as well as by strengthening existing networks in order to optimize the use of the continent’s scarce resources (African Union). It has been proposed that the AMA will also facilitate joint assessments and Good Manufacturing Practices (GMP) inspections for active pharmaceutical ingredients and products such as vaccines (African Union).

The AU Executive Council Decision EX.CL/Dec.857 (XXVI) of January 2015 forms the basis for the establishment of the AMA and endorsed the milestones for establishing the continental regulator within the context of the African Medicines Regulatory Harmonization (AMRH) initiative, which is a part of the implementation of the Pharmaceutical Manufacturing Plan for Africa (African Union, 2017; Ndomondo-Sigonda *et al*., 2018; Mukanga, 2019). The AMRH initiative was established in 2009 to address the regulatory challenges that national medicines regulatory authorities (NRAs) in Africa were grappling with. All countries in Africa (with the exception of Sahrawi Republic) have an NRA or an administrative unit conducting some or all expected NRA functions (Ndomondo-Sigonda *et al*., 2017). The core functions performed by African NRAs include marketing authorization, licencing of manufacturers, GMP inspections, market surveillance and clinical trials oversight (Ndomondo-Sigonda *et al*., 2017). However, >90% of African NRAs lack the capacity to perform the core regulatory functions. Most efforts are directed towards multiple sourced medicines as we note that even in South Africa only a minority of new originator medicines are being launched which may never reach the market as the dynamics would have changed since the initial application for marketing authorization (Mpanza *et al*., 2023). By comparison, regulators in high-income countries such as the European Medicines Agency and the United States Food and Drugs Administration mainly authorize innovative medicines, including for rare diseases, and facilitate their development as well as enable access to such products (European Medicines Agency; USFDA). Regulators on the African continent also have different WHO maturity levels, organizational structures and mandates. Some NRAs are semi-autonomous whereas others are housed in their Ministry responsible for Health, and some NRAs regulate medical products only whereas others have the mandate to also regulate food and cosmetics (Ndomondo-Sigonda *et al*., 2017). To alleviate the capacity constraints in Africa, a number of initiatives exist to reduce the regulatory burden for individual countries and rapidly bring quality-assured medical products to the market to benefit all key stakeholders. These initiatives include WHO Prequalification, Swissmedic Marketing Authorization for Global Health Products, the European Medicines Agency Medicines-4-All programme (Ncube *et al*., 2022), as well as regional work-sharing schemes such as Zazibona (Sithole *et al*., 2020), the East African Community (EAC) Medicines Regulatory Harmonization initiative (Arik *et al*., 2020; Mashingia *et al*., 2020), and the harmonization initiative of the Economic Community of West African States (Owusu-Asante *et al*., 2022). The AMRH initiative also exists to facilitate and coordinate the harmonization of medicines regulatory systems and to improve access to medicines on the continent. Access to medical products is enabled by the presence of functional and well-resourced NRAs.

The AMA’s scope of activities will include promotion of medical product policy, legal and regulatory reforms in African countries, development of technical guidelines and standards for NRAs to use, evaluating and assessing medical products regulatory systems in Africa in order to improve their efficiency and effectiveness, strengthening the capacity of NRAs through the provision of technical support and resources and resource mobilization to support regulatory systems strengthening efforts on the continent (African Union, 2017). The Agency’s activities, such as the evaluation of marketing authorization applications for complex medical products and GMP inspections of foreign manufacturing sites (African Union), will be performed by a small complement of competent staff and experts drawn from AU Member States (African Union, World Health Organization, 2014). As the AMA will only have an advisory and consultative role, NRAs will still have their decision-making roles and implement market controls for their respective territories (African Union). Therefore, NRAs and subregional medicines regulatory authorities will not be replaced by the AMA (African Union). AU Member States also have the responsibility of being the AMA’s primary source of funding, in addition to contributing in kind to the AMA’s work by dedicating part of the time of their NRAs’ staff (African Union, World Health Organization, 2014). Financial institutions and development partners can provide funding for the continental regulatory agency (African Union, World Health Organization, 2014).

The AMA was expected to be launched in 2018 (African Union Commission, World Health Organization, 2014; Luthuli and Robles, 2017), with efforts being made to ensure that the agency capitalizes on already existent mechanisms, experiences and technologies to work in an effective manner towards the accomplishment of its objectives (African Union Commission, World Health Organization, 2014). However, 5 years later, the AMA is not yet operational. Some of the factors that must be considered when operationalizing the AMA include its operational governance, financial sustainability, alignment of regulatory standards in Africa, and human resources and technical capacity. Ncube *et al*. (2022) have published on workforce capacity development and harmonization activities towards the establishment of the AMA. The AMA is being established by a treaty to effectively address some of the regulatory challenges that are being faced by African countries. These challenges include countries having noncoherent legislative frameworks, lengthy medicine registration processes, backlogs in reviewing marketing authorization applications, inadequate financial resources, gaps in the development of a unified regulatory science body and the lack of a competent regulatory workforce. For the AMA to be established, the AMA treaty (African Union, 2019b), which is open to AU Member States for signature and ratification/accession, had to be signed and ratified by a minimum of 15 AU Member States. The treaty then entered into force on 5 November 2021, 30 days after the 15th instrument of ratification was received by the AU Commission. The final form that the AMA will take is unclear (Ndomondo-Sigonda *et al*., 2020). However, it was decided that the AMRH initiative shall serve as the foundation for the establishment of the AMA (African Union; 2019b; 2020). The AMA is also expected to become Africa’s focus of regulatory standards harmonization, process optimization and resource coordination across the continent (Ndomondo-Sigonda *et al*., 2020). Furthermore, the AMA is envisaged to represent a single, credible African voice that has more weight compared with having individual voices on regulatory issues on the continent (African Union).

The treaty ratification process has been slower than expected and currently 20 out of 55 African countries have ratified the treaty and deposited their ratification instruments. The slow ratification process hinders harmonization and regulatory systems strengthening at a continental level. Without a stable, well-functioning and integrated regulatory system, Africans are therefore at risk of injury or death following the consumption of SF medical products. This was seen when at least 60 children, mostly under the age of five, died from acute kidney injury in The Gambia after consuming cough syrup contaminated with ethylene glycol and diethylene glycol (Bastani *et al*., 2023). The AMA can address the circulation of SF medical products in Africa by providing technical and strategic support to countries in order to improve vigilance functions and safety surveillance capacity. The AMA can also build on existing initiatives, such as the Lomé agreement (Brazzaville Foundation), the African Union Smart Safety Surveillance (AU-3S) (AUDA-NEPAD) and Pan-African regulatory collaborations conducted with the WHO. Ncube *et al*. (2021) recommended research to gain an understanding of the reasons for the slow uptake of signing and/or ratifying the AMA treaty. This will provide valuable insights into NRAs and AU Member States’ perceptions of the benefits they will accrue from the establishment of a continental regulatory body, the key internal and external actors in the ratification process that must be lobbied to ratify the treaty, as well as reservations about the AMA’s establishment. The aim of this study was therefore to analyse in depth the rationale, perceived benefits, enabling factors and challenges faced by AU Member States in the establishment of the AMA.

## Materials and methods

This study was a qualitative, cross-sectional, census survey of the NRAs of Anglophone and Francophone AU Member States. NRAs that do not actively participate in the AMRH initiative were excluded from the study as the contact details of the Head of the NRA or an AMRH initiative liaison were not available in the initiative’s database. These countries are Djibouti, Libya, Malawi, Mauritius, and Sahrawi Republic. Lusophone AU Member States (Angola, Guinea-Bissau, Mozambique and São Tomé and Príncipe) were also excluded from the survey due to the lack of capacity to translate the questionnaires and respondents’ responses from English to Portuguese and vice versa. Equatorial Guinea was excluded due to Spanish and Portuguese being the official languages. Furthermore, Rwanda was excluded from the main survey as the research instruments were piloted on the Rwanda Food and Drugs Authority. Rwanda FDA was conveniently sampled for the pilot study and the Director General of the Rwanda FDA and the Head of the Drugs and Food Assessment and Registration department were requested to complete the questionnaires.

The NRAs of the remaining 45 African jurisdictions were included in the study, and two regulatory officials, viz., the Head of the NRA and their Chief Regulatory Officer (or an alternative senior competent person) from each NRA, were purposively sampled and contacted via electronic mail to complete the questionnaire on Survey Monkey. The questionnaire was developed using the expertise of the authors. To achieve a content valid instrument, a rational analysis of the instrument is typically done by experts familiar with the construct of interest or experts on the research subject (Bolarinwa, 2015). Therefore, regulatory affairs and policy professionals from the International Federation of Pharmaceutical Manufacturers & Associations (IFPMA) African Regulatory Network and Temple University reviewed all the questionnaire items for clarity, comprehensiveness and readability. An agreement was then reached by the authors on which items should be included in the final research questionnaire. The questionnaire for this study consisted of mainly open-ended questions that elicited perceptions on the rationale and motivation for AMA treaty signing and ratification and the factors that enable and pose challenges to the process. Depending on the official language spoken in the recipient’s country, self-administered questionnaires, the accompanying information and consent documents were provided in either English or French. Two bilingual healthcare professionals (a medical doctor and a pharmacist) independently translated the questionnaires from English to French and sent these to B.M.N. who served as the language coordinator. The translators were briefed on what they are translating in order for them to know as much as possible about the study and context and translate accurately. The translation exercise aimed to achieve ‘pragmatic equivalence’ in translation instead of ‘semantic or conceptual equivalence’. Pragmatic equivalence aims ‘to have the same effect in the target language reader as the original would have in the source language reader’ (Ozolins *et al*., 2020). B.M.N. compared the translations and discussed any apparent discrepancies with the translators who provided their rationales for the choices they made. After the discussion and agreement on items, a final version of the questionnaire was developed and used in this research study.

Participants were given 6 weeks (between October and November 2021) to complete and submit the questionnaires, and four reminder emails with the Survey Monkey link were sent out during this period. In the Results section, ‘P’ refers to a study participant. As the survey involved high-level participants, AMRH initiative staff at the African Union Development Agency—New Partnership for Africa’s Development (AUDA-NEPAD) were engaged to support this research and facilitate access to NRAs in the data collection phase. The qualitative data were subjected to open coding and thematic analysis. Inductive analysis was done. Although this was a qualitative study, there were a few variables that were quantitative in nature. These data were summarized using descriptive statistics in Microsoft Excel. The study was approved by the Humanities and Social Sciences Research and Ethics Committee (HSSREC), University of the Western Cape, South Africa (HSSREC Reference Number: HS21/5/39).

## Results

Twenty-six completed questionnaires were received from 21 NRAs. Sixty-nine per cent (*n* = 18) of the questionnaires were from NRAs in Anglophone countries (Botswana, Ethiopia, Ghana, Kenya, the Kingdom of Eswatini, Liberia, Namibia, Seychelles, Sierra Leone, South Sudan, Tanzania (mainland), Tanzania (Zanzibar), The Gambia and Zimbabwe). The remaining 31% (*n* = 8) of the questionnaires were from NRAs in Francophone countries (Burundi, Cape Verde, Comoros Islands, Ivory Coast, Niger, Togo and Tunisia). No responses were received from Algeria, Benin, Burkina Faso, Cameroon, Central African Republic, Chad, Congo Republic, Democratic Republic of Congo, Egypt, Equatorial Guinea, Eritrea, Gabon, Guinea, Lesotho, Madagascar, Mali, Mauritania, Morocco, Nigeria, Senegal, Somalia, South Africa, Sudan, Uganda and Zambia. This study therefore had 47% of the NRAs participating in the research and a 29% response rate from the participating officials.

At the time of this study, 55% (*n* = 11) of the countries whose NRAs participated in this research had signed the AMA treaty. Of these, only 46% (*n* = 5) had also ratified the AMA treaty. Overall, the treaty had not been ratified by 75% (*n* = 15) of the countries whose NRAs participated in this research. All the NRAs in countries that had neither signed nor ratified the AMA treaty stated that their countries had an intention to do so.

The results in this section are organized using the order of questions from the questionnaire, and themes are introduced under each heading from the questionnaire. Sample participant quotes are provided to support the findings.

### The perceived advantages of the establishment of the AMA

All study participants perceived advantages to the establishment of the AMA. The AMA is perceived to enable reliance and recognition mechanisms to be implemented by African NRAs, enable access to medical products across the continent by supporting marketing authorization and serving as an advisory body for complex products such as biologicals, improve regulatory systems across Africa, develop NRAs’ regulatory capacities and expertise, as well as to enable regulatory harmonization on the continent. There is also a perception that the AMA’s establishment will create employment opportunities and incentives for pharmaceutical manufacturers to set up their factories in African countries.

### The perceived disadvantages of the establishment of the AMA

Eighty-one per cent of respondents (*n* = 21) stated that in their country there were no perceived disadvantages to the establishment of the AMA, while the remaining five respondents highlighted some disadvantages. One of the perceived disadvantages of operationalizing the AMA is that some duplication of regulatory effort may be present. Secondly, the scope and mandate of the AMA is perceived to be ambiguous and there is a fear of the AMA taking up the roles of NRAs. Additionally, there is a perception that once the AMA is established and countries sign and ratify the AMA treaty, financial contributions will be required from countries with existing overstretched budgets. Furthermore, there is a belief that the AMA will undermine national autonomy and reduce the revenue generated by national and regional authorities. Lastly, there is a *‘concern whether countries with very limited regulatory capacity will have a voice or will have their needs catered for within the institution. One may find that the organisation continues to cater more for those with greater regulatory capacity and those with lower maturity level continue to be left behind’* (P26).

### African NRAs’ expectations of the AMA

The AMA is expected to improve access to essential medical products as participants reported that *‘the AMA is in an important position to leverage several regulatory assets and resources to improve access to essential medicines and safe, effective, quality and affordable health technologies’* (P24). They also expect the AMA to *‘support market authorisation and improve quality of medicines distributed in the African Union’* (P13) and *‘approve medical products in the event of a health emergency’* (P23). In addition, the continental regulator should *‘provide advice on advanced therapies, such as biologicals, and the regulation thereof’* (P5).

The AMA is expected to be an information sharing agency, strengthen and harmonize regulatory systems on the continent, assist countries establish NRAs and build national regulatory capacity, as well as curb the circulation of SF medical products in Africa.

There is also an expectation that the AMA will *‘have a fair, transparent system and regional representativeness in the selection and appointment of experts/consultants’* (P16). The same participant further stated that there must be *‘transparent good practices in reaching decisions on recommending products’* and *‘independence from foreign governments and development partners with ulterior motives’* (P16). Moreover, respondents from the same country (P10 and P12) stated that they expect the AMA to be hosted in their capital city and the AMA should be an agency that creates employment opportunities.

### Perceptions of African NRAs’ roles and contributions to/in the AMA

African NRAs consider their role and contribution to/in the AMA to be active participation and involvement in all decisions, to avail any support particularly the technical expertise needed to carry out the AMA’s mission as well as to share data and information, including on SF medical products. Additionally, NRAs are keen to participate in strengthening pharmaceutical cooperation and to contribute to the assurance of safe, effective and quality medicines on the continent. Two participants (P7 and P22) from different countries mentioned that their contribution to/in the AMA will be based on successful participation in regional medicines regulatory harmonization initiatives and being an active player in the establishment of harmonized regulations within the African continent. NRAs also perceive their role to encompass financial support for the AMA, if necessary. In addition, there is hope that NRAs would be *‘consulted on the needs of member states with a lower maturity level’* (P26).

### The process to sign and ratify the treaty for the establishment of the AMA

**Figure 1.**
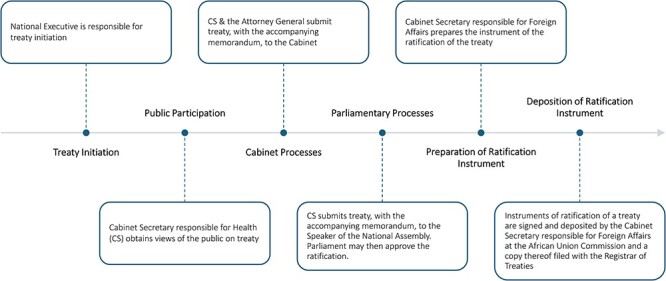
The common process followed to ratify the AMA treaty. The general responsibility for treaty initiation is the National Executive, and this may be delegated to a relevant State Department. In this regard, the CS responsible for Health is required to undertake public participation to seek and obtain the views of the public on the treaty. Upon undertaking public participation, the CS, in consultation with the Attorney General, is required to submit the treaty to the Cabinet together with the accompanying memorandum. Upon approval for ratification by Cabinet, the CS is required to submit the treaty and a memorandum on the same to the Speaker of the National Assembly. Parliament may then approve the ratification with or without reservations to specific provisions of the treaty. Where ratification is approved without any reservations to the treaty, the CS shall, within 30 days from the date of approval of the ratification of the treaty, request the CS responsible for Foreign Affairs, to prepare the instrument of the ratification of the treaty. However, if there is approval with reservations, the treaty shall be ratified with those reservations to the corresponding article in the treaty. If it is refused by Parliament, the Government shall not ratify the treaty. All instruments of ratification of a treaty shall be signed and deposited by the CS responsible for Foreign Affairs at the AU Commission and a copy thereof shall be filed with the Registrar of Treaties. The Ministry responsible for Health in the country is also obliged to take measures to inform and create awareness to the public about the effects and benefits of the treaty

The processes undertaken in signing the treaty vary between AU Member States. However, several countries follow a process similar to the one described by P12 and outlined in Figure 1. An alternative process described by a participant (P8) involves the treaty needing to *‘first be signed by the President or an authorised representative of the President. The treaty must then be submitted to Parliament for ratification either by an Act of Parliament or by a resolution of Parliament supported by the votes of more than one half of all the members of Parliament. After this process is completed, the instrument of accession is deposited to the African Union Commission’*. In another country, *‘the NRA wrote a concept note to the Minister of Health, who then has the responsibility of writing to the Minister of Foreign Affairs requesting the latter to submit a memo to the Council of Ministers to approve the process to sign and ratify the AMA treaty. The Minister of Justice then presents the request to the National Legislature for endorsement and approval’* (P3).

The process can also start with the Minister responsible for Foreign Affairs signing the treaty, after which the Minister of Health tables the treaty in Parliament and if it is approved, it is published in the government gazette and then implemented (P9). Participant P10 outlined a process that begins with the drafting of a concept note which is sent to the Ministry of Health for approval. The Ministry of Health then takes the matter to Cabinet for discussion and development of a Cabinet Memo. Next, the Parliament endorses/ratifies the treaty, and the Cabinet Secretary (CS)—Ministry of Foreign Affairs approves and forwards the ratification instrument to the AU Commission. (P10)

Two participants (P13 and P26), both from smaller African states, stated that they were not sure of the process.

### Facilitators, advocates or champions in the process of signing and ratifying the AMA treaty

Facilitators, champions or advocates for the signing and ratification of the AMA treaty can either be internal, that is within the NRA, or external.

Fifty-five per cent (*n* = 11) of countries reported that they had internal facilitators, and 60% (*n *= 12) reported that they had external facilitators. Some countries had both internal and external facilitators.

In terms of internal facilitators, the Head of the NRA was most commonly cited along with the NRA’s Board. Their roles involved *‘advocacy at the ministerial level’* (P19), *‘spearheading the signing and ratification of the treaty’* (P8), as well as preparation of the Bill and explanatory memorandum/concept notes. Other internal facilitators include NRA staff, including the legal departments and committees, pharmacists, the NRA’s focal person for the regional medicines regulatory harmonization initiative and the NRA’s Chief Regulatory Officer.

The most frequently mentioned external facilitators, advocates or champions in the signing and ratification of the AMA treaty are the Minister of Health, AUDA-NEPAD and Honourable Michel Sidibé, the AU Special Envoy for the AMA. According to respondents, the Minister of Health facilitated the process and supported NRAs with communication and advocacy to government to ratify the treaty. AUDA-NEPAD and the Special Envoy played an advocacy role, providing information about the AMA to NRAs and governments, and Honourable Michel Sidibé also made courtesy visits to some AU Member States to encourage the leadership to sign and ratify the treaty (P16). Other external facilitators include regional economic communities (RECs) [namely the Southern African Development Community (SADC) and the EAC], Permanent Secretaries for Health, the AU and the AU Commission, the WHO African Regional Office and WHO country offices, the Ministry in charge of Foreign Affairs, the Parliamentary Select Committee on Health, the Cabinet, PATH (the United States based nonprofit global health organization), Amref Health Africa and the pharmaceutical industry.

### Enabling factors for the signing of the treaty for the establishment of the AMA

Having an established NRA is considered to be an important enabling factor for the signing of the AMA treaty by an AU Member State as well as having a robust regulatory system. Another factor that participants consider to be important is the desire of their NRAs and/or Ministries responsible for Health to have harmonized regulatory systems in Africa that allow for collaboration. In addition, there must be strong political will and support from the Ministry responsible for Health and the government as well as appropriate advocacy to expedite treaty signing. Furthermore, the presence of internal facilitators, advocates or champions in the NRA enables treaty signing, especially when the advocates are the Heads of the NRAs, and they have *‘adequate awareness of the AMA treaty’* (P9). One participant (P16) also stated that the *‘active participation of [their NRA’s] former Director-General in the AMA Steering Committee’* served as a facilitator of the treaty signing process. Moreover, there must be technical and financial resources to support the treaty signing process and *‘technical and financial support from external parties such as AUDA-NEPAD, WHO and other donor organisations’* (P8). According to P20, *‘the creation of the African Continental Free Trade Area’* in their country served as an enabling factor for the signing of the AMA treaty.

### Challenges or barriers encountered in signing the treaty for the establishment of the AMA

Most of the participants from countries that have signed the treaty stated that there were no challenges or barriers encountered. The most frequently cited challenge is the tedious nature of the process. Participants also reported that there is a lack of awareness and limited understanding of the signing of the treaty. Additionally, in some countries, *‘the bureaucracy and red tape’* (P12) present a challenge and it is difficult to convince their leaders to sign. Competing national priorities, administrative and legislative procedures, changes in office bearers in the public system and stagnation of the process at the ministerial level are also challenges encountered. One participant (P17) mentioned that they are *‘keen to participate but there is little support in the processes that will follow’*.

To overcome these challenges or barriers, NRAs were *‘pushing the concerned authorities to speed up the process’* (P3), calling for *‘more political involvement’* (P19) and conducting *‘more advocacy for the Ministers of Health, Foreign Affairs, Justice, and the Government’*. They also improved their follow-up of documentation at the Ministry responsible for Health for the process to proceed to the ratification stage. Dialogue and stakeholder engagement featured prominently as an advocacy strategy to create awareness among key stakeholders. Furthermore, some African NRAs enlisted *‘strong external advocacy from international institutions (i.e. SADC, AU, and WHO)’* (P18) to persuade the government to sign and ratify the treaty.

## Discussion

This research focused on the signing and ratification of the AMA treaty which serves to create a supranational regulatory authority. The AMA is believed to have the unique opportunity to become one of the most efficient and modern regulatory systems in the world (International Federation of Pharmaceutical Manufacturers & Associations (IFPMA)). This opportunity can quickly become reality by using the experience acquired over the last 10 years of harmonization activities in Africa, lessons learned during the coronavirus disease 2019 pandemic as well as the expedited implementation of modern and innovative solutions (International Federation of Pharmaceutical Manufacturers & Associations (IFPMA)). A number of attempts at supranationalism have been made in Africa over the years, and some of these attempts have succeeded while others have failed (Fagbayibo, 2010).

Drawing lessons from previous supranationalism successes, African states participate in regional integration initiatives due to the desire to accrue immediate-to-long-term benefits; however, when these benefits do not materialize, it is likely that the country will either partially commit to the objectives of integration or completely pull out (Fagbayibo, 2010). In this study, enabling access to medical products across the continent is considered a benefit of establishing the AMA. Access to medicines is an area of concern as there is typically a lag of 4 to 7 years between first regulatory submission, often in a high-income country, and final approval of the medical product in sub-Saharan Africa (Ahonkhai *et al*., 2016). This is one factor leading to poor access. Secondly, new originator medicines can account for only 13% of registered medicines and up to 27% of medicines granted marketing authorization by an African NRA can take 5 years from the year of registration to reach patients in private community pharmacies (Mpanza *et al*., 2023). Affordability is also a barrier to access. For example, trastuzumab which is used in breast cancer management in Africa was available in 10 out of 19 facilities across 14 African countries; however, only 5% of patients could afford it (Vanderpuye *et al*., 2017). Gershon *et al*. (2019) also found trastuzumab to not be cost effective in 11 African countries studied, and Al-Ziftawi *et al*. (2021) reported similar findings. Registration and use of generics is a strategy used to lower medicine costs and improve access in sub-Saharan Africa (Koduah *et al*., 2022). The same strategy has been used in Europe where the expenditure for generic omeprazole and simvastatin decreased in The Netherlands despite at least a 3-fold increase in utilization, enabled by the generics’ increased use at 2% of pre-patent loss prices (Woerkom *et al*., 2012). Another perceived benefit of establishing the AMA is that the continental agency will enable regulatory harmonization in Africa. As it stands, five out of the eight RECs in Africa (the EAC, Economic Community of Central African States, Economic Community of West African States, Intergovernmental Authority on Development and SADC) have medicines regulatory harmonization initiatives, although at different maturity levels. These regional harmonization initiatives provide evidence that the perceived benefits reported by participants will materialize as they have accrued these benefits and the AMA is meant to build on these initiatives.

In Africa, there is typically a considerable difference between commitment to supranationalism at public fora and the creation of an environment that is conducive for operational success (Fagbayibo, 2010). This is attributed to the lack of political will on the part of African governments to bring goals and objectives to fruition (Thompson, 1990). Lack of political will has in the past hindered attempts at supranationalism on the continent regardless of the legal frameworks used in the country (Kufuor, 1996). In this study, a chief enabler for signing and ratifying the AMA treaty was the presence of political will, which was also noted in successful ratification of the African Continental Free Trade Area (AfCFTA) agreement (Muftau). The AfCFTA aims to create a single market for the continent, enabling the free flow of goods and services and boosting the trading position of Africa in the global market (African Union). The AfCFTA agreement entered into force on 30 May 2019 for the 24 AU Member States that had deposited their ratification instruments by this date (Tralac). The operational phase of the AfCFTA was then launched on 7 July 2019 during the 12th Extraordinary Session of the Assembly of the Union on the AfCFTA in Niamey, Niger (Tralac). 1 January 2021 marked the start of trading under the agreement (Tralac).

The majority of participants in this study failed to raise any concerns regarding the establishment of the AMA. However, some participants reported that the scope and mandate of the AMA is ambiguous. Similarly, Nigeria initially did not sign the AfCFTA agreement due to the perceived uncertainty about the benefits of the AfCFTA to the country (Muftau). There are also concerns about the AMA undermining national autonomy and fears of the continental regulator taking over the roles of NRAs. Muftau contends that there is a pattern that repeats itself, even with the AfCFTA, of African countries refusing to cede sovereignty to supranational bodies which results in the adoption of intergovernmental entities that have limited authority to bind state parties as well as slow or non-implementation of treaty obligations. Furthermore, there is the possibility of duplicating regulatory efforts once the AMA is operationalized. Duplication of effort could be a valid concern due to some countries such as Tanzania, being a member of two RECs—the EAC and SADC—thus requiring active participation from NRAs. Thus, with the inception of the AMA, NRA staff may be expected to lend expertise to a national regulator, two regional medicines regulatory harmonization initiatives and a continental regulatory authority. Literature supports this finding, and scholars argue that having multiple RECs with overlapping and replicated membership has contributed to failures to integrate and establish supranational institutions (Fagbayibo, 2010). This is due to the RECs lacking unity in their approaches and goals. This, however, is not the reason for duplication. Countries join more than one REC for strategic reasons such as having a decision-making role in different regions in Africa (Fagbayibo, 2010).

The expectation of financial contribution towards the establishment and operation of the AMA was also raised as a concern in this study. Many low- and middle-income countries cannot finance their public health needs, and their NRAs are particularly vulnerable (Roth *et al*., 2018). Currently, African governments are failing to meet health financing targets. In April 2001, AU Member States met in Abuja, Nigeria, where they committed to allocate 15% of their government budgets to health (Gatome-Munyua and Olalere, 2020). This commitment is referred to as the ‘Abuja Declaration’, and in any given year since 2001, only a handful of African countries have met this target (e.g. only two countries met the target in 2018) (Gatome-Munyua and Olalere, 2020). Due to underperforming economies which result in some AU Member States being unable to meet their financial obligations to the regional organizations that they belong to, the establishment of supranational organizations outside of medical products regulation has been unsuccessful (Fagbayibo, 2010). The AMA will therefore require sustainable financing mechanisms to enable it to operate successfully.

Concerns were raised about whether countries with very limited regulatory capacity will be treated equitably with respect to decision-making in and receiving support from the AMA. The concern of study participants is that the AMA, once operational, may favour countries with greater regulatory capacity. This may be a valid concern as regional integration has often resulted in African countries with stronger economies accruing maximum benefit from the integration initiatives to the detriment of other member states (Fagbayibo, 2010). Moreover, study participants fear that countries with lower (WHO) maturity levels will continue to lag behind with respect to regulatory agency development. This fear creates a barrier to establishing supranational institutions as smaller AU Member States consequently withdraw support from regional organizations that are believed to be *‘the mouthpiece’* of regional powers (Fagbayibo, 2010). These regional organizations subsequently lose legitimacy (Fagbayibo, 2010).

Competing national priorities, administrative and legislative procedures, changes in office bearers in the public system and stagnation of the process at the ministerial level are other challenges encountered. Scholars of supranationalism report that regional institutions in Africa are expected to function based on *‘the whims of member states […] rather than to fulfil the ambitious objectives of the organisation’* (Fagbayibo, 2010). As a result, there are examples in literature of integration initiatives being derailed or dissolved following changes in administration in member states or due to personal difference among Heads of States and Governments (Fagbayibo, 2010). The lack of independence of regional institutions therefore impedes the implementation of integration initiatives (Fagbayibo, 2010).

The perceived disadvantages and challenges of establishing the AMA outlined in the previous paragraphs may be the reasons for the slower than anticipated processes towards AMA treaty ratification and for the lack of ratification in some countries with superior regulatory systems This study found that the process to sign and ratify the AMA treaty differs between countries, and this is due to treaty ratification having to occur within the confines of a national legal system which can either be monist or dualist. In monist countries, international law is directly applied in the country, whereas in dualist countries, treaties are not applied in the country unless they are incorporated through legislation. Generally, monist legal systems are used in civil law countries in Africa and dualist legal systems are used in common law countries (Ekhator, 2021). Former British colonies inherited the English legal system (common law), and previous French, Spanish and Portuguese colonies inherited the Napoleonic system (civil law) (Joireman, 2001). There does not seem to be a significant improvement in the treaty signing or ratification process when one of the legal systems is in force instead of the other. Research should, however, be conducted to investigate whether the legal differences alter the way in which ratification is achieved in different countries or if they have any implications for the future operation of the AMA.

Based on the findings of this study, the following recommendations are made:

- In countries that are yet to sign and/or ratify the AMA treaty, facilitators, advocates and champions must be identified and equipped with information and resources to advocate for these processes to occur. The AU, AUDA-NEPAD, RECs and development partners must continue to lobby and encourage AU Member States that are yet to sign and/or ratify the treaty to expedite their internal processes in order for the AMA to have membership from all African countries. NRAs and patient organizations should be involved in the advocacy work and decision-making processes as they are key stakeholders that can enable the process.
- The AMA’s shape, role and governance structure should be clearly stated and communicated to NRAs and AU Member States in order to address any ambiguities about the scope and mandate of the AMA.
- The AMA should have sustainable financing mechanisms that do not place a burden on member states and NRAs.
- The AMA should have a fair, transparent system and regional representativeness in the selection and appointment of experts/consultants. It should also equitably cater for the needs of small countries and countries with limited regulatory capacity.
- Once the AMA is operational, there should be no duplication of effort by NRA, regional regulatory harmonization initiatives and the continental regulator.
- The AMA should foster an environment that is conducive for innovation and the biopharmaceutical industry in order to improve access to quality-assured, safe and efficacious medical products for Africans.

## Study limitations

It is possible that the experiences of the countries that were excluded from the study are different from the included study participants. Another limitation concerns the lack of triangulation in this study and failure to compare the AMA treaty ratification process to processes in other territories such as the European Union.

## Conclusion

This study analysed the rationale, perceived benefits, enabling factors and challenges of the establishment of the AMA. It found that the existence of mature NRAs, the desire to have harmonized regulatory systems in Africa that allow for collaboration, the presence of strong political will and support from the government as well as appropriate advocacy to expedite treaty signing are all enabling factors for the signing of the AMA treaty. The challenges encountered in treaty signing include the process being slow and a lack of awareness and limited understanding by African leaders of the treaty signing process. Competing national priorities, administrative and legislative procedures and changes in office bearers in the public system are also challenges encountered. Overall, there is strong support for the establishment of a continental regulator that represents a single credible African voice.

## Data Availability

The data underlying this article will be shared on reasonable request to the corresponding author.

## Abbreviations

AMA = African Medicines Agency

AMRH = African Medicines Regulatory Harmonization

AU = African Union

AUDA-NEPAD = African Union Development Agency—New Partnership for Africa’s Development

EAC = East African Community

GMP = Good Manufacturing Practices

NRA = national medicines regulatory authority

RECs = regional economic communities

SADC = Southern African Development Community

## Reflexivity statement

This paper was authored by two males and one female with a range of experience and seniority. The authors are all residents in Southern Africa. The three authors have conducted research and published work together on the implementation of the African Union Model Law on Medical Products Regulation and the establishment of the AMA in international peer-reviewed journals.
